# Theoretical and empirical dimensions of the Aberdeen Glaucoma Questionnaire: a cross sectional survey and principal component analysis

**DOI:** 10.1186/1471-2415-13-72

**Published:** 2013-11-22

**Authors:** Maria Prior, Craig R Ramsay, Jennifer M Burr, Susan E Campbell, David J Jenkinson, Ryo Asoaka, Jillian J Francis

**Affiliations:** 1 Health Services Research Unit, University of Aberdeen, Aberdeen, UK; 2 School of Medicine, University of St. Andrews, St. Andrews, Fife, UK; 3 School of Nursing Sciences, University of East Anglia, Norwich, UK; 4 School of Health & Population Sciences, University of Birmingham, Birmingham, UK; 5 NIHR Biomedical Research Centre, Moorfields Eye Hospital NHS Foundation Trust and University College London Institute of Ophthalmology, London, UK; 6 Department of Optometry and Visual Science, City University London, London, UK; 7 Department of Ophthalmology, University of Tokyo Graduate School of Medicine, Tokyo, Japan; 8 School of Health Sciences, City University London, London, UK

## Abstract

**Background:**

To develop patient-reported outcome instruments, statistical techniques (e.g., principal components analysis; PCA) are used to examine correlations among items and identify interrelated item subsets (empirical factors). However, interpretation and labelling of empirical factors is a subjective process, lacking precision or conceptual basis. We report a novel and reproducible method for mapping between theoretical and empirical factor structures. We illustrate the method using the pilot Aberdeen Glaucoma Questionnaire (AGQ), a new measure of glaucoma-related disability developed using the International Classification of Functioning and Disability (ICF) as a theoretical framework and tested in a sample representing the spectrum of glaucoma severity.

**Methods:**

We used the ICF to code AGQ item content before mailing the AGQ to a UK sample (N = 1349) selected to represent people with a risk factor for glaucoma and people with glaucoma across a range of severity. Reflecting uncertainty in the theoretical framework (items with multiple ICF codes), an exploratory PCA was conducted. The theoretical structure informed our interpretation of the empirical structure and guided the selection of theoretically-derived factor labels. We also explored the discrimination of the AGQ across glaucoma severity groups.

**Results:**

656 (49%) completed questionnaires were returned. The data yielded a 7-factor solution with a simple structure (using cut-off point of a loading of 0.5) that together accounted for 63% of variance in the scores. The mapping process resulted in allocation of the following theoretically-derived factor labels: 1) Seeing Functions: Participation; 2) Moving Around & Communication; 3) Emotional Functions; 4) Walking Around Obstacles; 5) Light; 6) Seeing Functions: Domestic & Social Life; 7) Mobility. Using the seven factor scores as independent variables in a discriminant function analysis, the AGQ scores resulted in correct glaucoma severity grading of 32.5% of participants (p < 0.001).

**Conclusions:**

This paper addresses a methodological gap in the application of classical test theory (CTT) techniques, such as PCA, in instrument development. Labels for empirically-derived factors are often selected intuitively whereas they can inform existing bodies of knowledge if selected on the basis of theoretical construct labels, which are more explicitly defined and which relate to each other in ways that are evidence based.

## Background

Measures that reflect patients’ assessment of their health are increasingly important as outcome measures in both clinical practice and research. The decision whether to develop a new patient reported outcome (PRO) instrument or use an existing validated measure should be based on a thorough review of PRO instruments used in a population of interest [1,2]. If a new instrument is required, robust and transparent methods should be used at every stage of its development.

We have previously reported the systematic pre-validation development of a pilot PRO – the Aberdeen Glaucoma Questionnaire (AGQ), a new measure designed for use in a research context to compare vision-related disability between intervention (glaucoma screening) and comparator (opportunistic case detection) arms at the end of a proposed RCT [3]. The development of the AGQ followed a systematic review of PRO instruments used in glaucoma populations, which concluded that none of the existing instruments was suitable for use in the proposed RCT [2]. The pre-validation development of the AGQ used the World Health Organisation (WHO) International Classification of Functioning and Disability (ICF) [4] as a theoretical framework [2,3]. This process involved using the ICF to code the AGQ item content [3,5] by: identifying *meaningful concepts* (i.e. ideas or information) contained within each AGQ item; linking each meaningful concept to the most precise ICF category.

Following the pre-validation development of new instruments such as the AGQ, the next step is to conduct psychometric analyses of the pilot instrument in the target population in order to assess the underlying structure [6]. Statistical techniques from classical test theory (CTT), such as principal components analysis (PCA), continue to be widely used in the initial stages of instrument testing to examine patterns of correlations among items and identify interrelated item subsets (empirical factors) [7,8]. Following the application of such methods, each identified empirical factor is assigned a descriptive label to indicate the traits it is hypothesised to measure (e.g. near and distance vision) [7].

However, the interpretation and labelling of empirical factors is a subjective process lacking precision or conceptual basis [9]. In this paper we report a novel and reproducible method for mapping between theoretical and empirical factor structures. In order to illustrate the method we present results from the early stages of testing the pilot AGQ in a population representing the spectrum of glaucoma severity, from those with a risk factor for the disease (i.e. ocular hypertension) to those with diagnosed glaucoma across a range of severity. The main objectives of this paper are to report: the empirical factors (‘content’ domains) identified within the AGQ; explicit methods for linking the empirical factors with the theoretical (ICF) factor structure and for assigning theoretically informed labels. A secondary objective is to report findings from the exploration of the discriminative ability of the AGQ across glaucoma severity.

## Methods

We used a cross-sectional postal survey design to test the pilot AGQ among patients, who had undergone Humphrey threshold visual field testing between January 2007 and September 2009. Potential participants were identified, by collaborating ophthalmologists, from a visual field database of a UK Hospital based glaucoma service (Moorfields Eye Hospital (MEH)). To be included a patient had to have at least one visual field entry in the database prior to the most recent and in addition a reproducible defect as determined by the Glaucoma Hemifield Test (a feature of the Progressor software; Moorfields Eye Hospital/Medisoft Ltd).

The postal questionnaire included four sections: 1) the pilot AGQ; 2) a generic health status measure - EQ-5D [10]; 3) questions relating to baseline demographic characteristics; 4) a widely used validated vision-status measure - the 25-item National Eye Institute Visual Function Questionnaire (NEI-VFQ 25) [11], which was included as a benchmark for comparison with the pilot AGQ. The results presented below focus on the pilot AGQ and on addressing the study objectives. Accordingly, we limit our reporting of results from sections 2, 3, and 4 to sample characteristics.

The pilot AGQ consisted of 68 items [3]. Thirty-nine items used a 4-point response option (e.g. No = 1, Sometimes = 2, Often = 3, Always = 4). A further 8 items contained an additional response option (e.g. unable to perform an activity = 5). These ordinal values were then used as real values for the PCA. Seventeen items used a dichotomised response option (No = 1, Yes = 2), with a further 4 items containing a third option to enable participants to indicate uncertainty (see Additional file 1: the AGQ). Throughout the pilot AGQ high scores indicate poor outcomes (e.g. functional impairments or difficulty performing activities). ‘Not applicable’ responses were coded as missing values.

The questionnaire was mailed to 1349 patients, who met the inclusion criteria, in March 2010 together with an information sheet, a letter of invitation from their MEH ophthalmologist and a reply paid envelope. One reminder letter was sent to non-responders two weeks later. No further contact was made with non-responders. The return of a completed questionnaire was considered as consent to take part. Ethics committee approval was obtained for the study from the North of Scotland Research Ethics Committee (Ref: 09/S0802/107). For those returning the questionnaire, we obtained data from the visual field databases on visual field parameters, mean defect and pattern standard deviation. Visual field loss in people’s *better* eye is indicated to have a greater impact on health related quality of life than does visual field loss in the *worse* eye [12]. In the analyses we used the most recent visual field data for each participant’s better eye.

### Analytical strategy

#### Identifying the empirical factors

Any items with >5% missing data were excluded from the analysis. The remaining missing data were not imputed. We conducted an exploratory PCA with Varimax rotation using a cut-off point of 0.5 on the rotated loadings [13]. The use of this approach reflected the uncertainty in the theoretical structure derived during the pre-validation development. This uncertainty resulted from the breadth of coverage of ICF components in the pilot AGQ and within each item (cross-coding on ICF components) [14]. The PCA identified the factors represented coherently in the data (empirical factors) to inform the sub-scale structure of the AGQ.

### Reliability

The internal consistency of each identified factor was assessed using Cronbach’s alpha [15]. For factors with Cronbach’s alpha coefficients of <0.7 we explored possible item inclusion/reduction as a strategy for improving internal consistency [9].

### Linking empirical factors with the ICF structure and label accordingly

The ICF uses an alphanumerical system in which the letters *b*, *d*, and *e* denote Body Functions (b), Activities and Participation (d) and Environment Factors (e) [4]. These letters are followed by numeric codes that range from one digit (to denote item content at the least specific (domain) level) to four digits (denoting highly specific attributes within each domain) (see Table 1).

**Table 1:** Example of the ICF hierarchical alphanumeric coding system

**ICF code**	**ICF heading for each code**
d5	Self-care
d540	Dressing
d5403	Taking off footwear

Table 2 provides an example AGQ item and the ICF coding for that item (assigned during the pre-validation development of the pilot AGQ). In order to identify theoretically robust, rather than intuitive, factor labels we mapped the *a priori* theoretical structure (ICF coding for each item) to the empirical factor structure. Within each factor, we examined the ICF code for each item and assigned factor labels that represent the highest level of specificity of ICF code applicable to each group of items.

**Table 2:** **Example of ICF coding rules applied during pre-validation development of the pilot AGQ [**[16]**]**

**Example AGQ item**	**ICF code**
How much does your *eyesight* interfere with your *getting about outdoors*? (on the pavement or crossing the street)	*b210 seeing functions d4602 moving around outside the home & other buildings*

### Discrimination of the AGQ across glaucoma severity

We graded patients according to four levels of glaucoma severity (ocular hypertension, mild, moderate and severe) using the mean defect (MD) of the better eye [12]. The grading interval cut-offs were: MD ≥ 0 (ocular hypertension); 0 > MD > -6 dB (mild glaucoma); -6 dB > MD > -12 dB (moderate glaucoma); and MD ≤ -12 dB (severe glaucoma). For an exploratory analysis we also graded patients according to the MD in the worse eye using the same criteria.

We conducted a breakdowns analysis using descriptive statistics to explore the distribution of summary AGQ scores by glaucoma severity in the better eye. In addition, Spearman’s rho correlation coefficients were calculated to formally represent the correlations between each AGQ factor and glaucoma severity. Finally, we performed a discriminant function analysis using leave-one-out cross validation, in SPSS v20 for Windows, on all seven AGQ factor scores to assess the extent to which AGQ scores could discriminate between groups formed on the basis of independently assessed glaucoma severity (in the better eye) [12]. The statistical significance was assessed using the Wilks’ lambda statistic in discriminant function analysis.

## Results

### Sample characteristics

Of the 1349 mailed questionnaires, 656 (49%) completed questionnaires were returned. The mean age of responders was 67.3 years (SD 13.3), 350 (53%) were female and 402 (61%) had mild glaucoma (0 > MD > -6 dB) based on the severity of the visual field loss in their better eye. The NEI-VFQ 25 and EQ5D scores by glaucoma severity are presented in Table 3 and indicate that the scores decrease (i.e. vision and health status worsen) as severity of glaucoma increases.

**Table 3:** Glaucoma severity grading (better eye) of respondents

	**n**	**%**	**Mean age (yrs)**	**NEI-VFQ 25 Mean score (SD)**	**EQ-5D Mean score (SD)**
Ocular hypertension *(normal)*	123	18.8	64.2	92.1 (7.50)	0.87 (0.20)
Mild	402	61.3	66.7	88.3 (11.67)	0.84 (0.20)
Moderate	82	12.5	72.1	79.1 (19.32)	0.79 (0.25)
Severe	49	7.5	72.8	68.6 (19.74)	0.80 (0.26)

### Identifying the empirical factors

Twenty-one items in the 68 item pilot AGQ were considered unreliable (due to >5% missing data) and were excluded from the PCA. Thirteen of these items related to local or systemic symptoms of glaucoma. The remaining eight excluded items were preceded by filter questions; the response to which determined whether participants should answer or skip subsequent items.

Using an eigenvalue cut-off of 1 [17] the data from the remaining 47 items yielded 7 factors that together accounted for 61% of the variance in the scores. Using a stringent cut-off point of a loading of 0.5 for item inclusion [13], 30 of the 47 items loaded on to a factor and presented a simple structure, (i.e. no items loaded on to more than one factor). Table 4 presents the item loadings.

**Table 4:** AGQ item loadings

**N = 656**							
**ITEM**	**F1**	**F2**	**F3**	**F4**	**F5**	**F6**	**F7**
Does your eyesight interfere with your recognising or meeting people?	0.58						
Do you have difficulty watching television? (appreciating the pictures)	0.60						
Do you have difficulty reading subtitles for film or TV?	0.62						
Do you have difficulty reading traffic signs, street signs, or store signs?	0.52						
Do you tend to confuse colours?	0.56						
Do you have difficulty finding something on a crowded shelf?	0.58						
How much does your eyesight interfere with using public transport on your own? (for instance bus, train or plane)		0.59					
Because of my eyesight I need help from family or friends.		0.64					
Because of my eyesight I have to rely on what other people tell me.		0.53					
Because of your eyesight, do you have difficulty going out of your home alone?		0.74					
Do you use assistance to get around? (e.g. a guide dog, cane, companion)		0.60					
Do you worry about your eyesight getting worse?			0.68				
Does your eyesight make you concerned or worried about coping with everyday life?			0.62				
Do you feel like a nuisance or a burden because of your eyesight?			0.61				
Do you feel embarrassed because of your eyesight?			0.60				
Do you feel frustrated or annoyed because of your eyesight?			0.66				
Because of your eyesight do you bump against other people in crowded areas?				0.70			
Do you bump into people or objects while walking?				0.72			
Do you trip over objects?				0.50			
Does your eyesight deteriorate in bright light?					0.67		
Does your eyesight deteriorate in dim light?					0.61		
Do you have difficulty with walking down steps in dim light?					0.54		
Do you have difficulty adjusting from bright to dim light? (such as when going from daylight into a dark room)					0.72		
Do you have difficulty with adjusting to bright lights?					0.72		
Because of my eyesight I need help from care services.						0.66	
Because of your eyesight, do you have difficulty entertaining friends and family in your home?						0.50	
When pouring liquid, do you have difficulty judging the level of the liquid in a container, such as the level of a cup of coffee?						0.55	
Do you have difficulty seeing how people react to things you say?						0.61	
In the last 12 months have you been anxious or worried about falling? (This may or may not be associated with a feeling of unsteadiness)							0.65
Have you fallen in the last 12 months?							0.71
Do you have difficulty with walking on uneven ground?							0.45^a^
Eigenvalue	19.67	2.09	1.69	1.49	1.35	1.14	1.06
Percentage variance explained	41.8	4.4	3.6	3.2	2.9	2.4	2.3

Notes: ^a^Item added to increase internal consistency.

### Reliability

Factor 7 initially contained only 2 items and the Cronbach’s alpha coefficient was <0.7. After examining the other items loading onto Factor 7, which did not meet the 0.5 cut-off, we included the next highest loading item (*Do you have difficulty with walking on uneven ground?*; item loading 0.45). The internal consistency of the 3-item Factor 7 improved as a result. Table 5 presents the summary statistics and internal consistency coefficients of the 7 factors.

**Table 5:** Summary data for the seven AGQ factors

**Factor label**		**Mean (SD)**	**Median (IQR)**	**Cronbach (α)**	**No. of items**
1	Seeing Functions: Participation	1.4 (0.50)	1.2 (0.50)	0.86	6
2	Moving Around and Communication	1.2 (0.40)	1.0 (0.20)	0.85	5
3	Emotional Function	1.5 (0.51)	1.4 (0.40)	0.84	5
4	Walking Around Obstacles	1.3 (0.46)	1.0 (0.33)	0.81	3
5	Light	1.8 (0.66)	1.6 (1.00)	0.86	5
6	Seeing Functions: Domestic & social Life	1.1 (0.31)	1.0 (0.00)	0.78	4
7	Mobility	1.4 (0.47)	1.3 (0.67)	0.68	3

### Items excluded after the PCA

Sixteen (of the 47) items in the PCA did not load onto any of the seven empirical factors (using a cut-off of 0.5) including all four items which, during the *a priori* theoretical ICF coding, were assessed to contain content that was too general to assign specific codes (i.e. items A5, A8, A25, A37) (see Additional file 1: the AGQ). A check on the 16 excluded items (i.e. item loadings <0.5) also showed that the content coverage of these items was adequately represented in the 7-factor solution. For example, the item *do you have difficulty walking in dimly lit indoor areas?* was excluded from the 7-factor solution, but this item contains content covered in the items of Factors 4, 5 and 7.

### Linking and labelling empirical factors with ICF structure

Table 6 presents the 31 items included in the 7-factor solution alongside the ICF codes assigned to each of these items during the development of the AGQ [3]. The juxtapositioning of the theoretical and empirical factor structure facilitated the allocation of theoretically derived factor labels. For example, in Factor 4, the empirical data suggest a label of ‘*bumping and tripping'*, whereas mapping back to the ICF theoretical structure highlighted the consistency on item content associated with ‘*walking around obstacles*’. In some factors, the level of specificity of item content varied. For example in Factor 7 all three items contain the ICF alphanumeric code d4 (mobility), but two of the items contain highly specific content in the d4 domain (i.e. ICF codes d410-d429, d4502). In such cases, factor labels reflect the highest level of specificity in common to all items, which for Factor 7 was mobility. In other factors, the item content differed not only by specificity, but also by content domain. In these cases, factor labels represent the combination of item content. This is illustrated by Factor 6 in which the content of its four items is represented by the label *seeing functions: domestic and social life* (i.e. *b210 seeing functions, d6 domestic life, d9 social life*). This mapping process resulted in the seven theoretically-derived factor labels of: 1) Seeing Functions: Participation, 2) Moving Around and Communication, 3) Emotional Function, 4) Walking Around Obstacles, 5) Light, 6) Seeing Functions: Domestic and Social Life, 7) Mobility.

**Table 6:** Mapping process for labelling the 7 empirical factors

**Factor no.**	**AGQ item content**		**ICF codes assigned to items during pre-validation phase (theoretical structure)**		**Factor label**
**Factor 1**	Does your eyesight interfere with your recognising or meeting people?		b210 ‘seeing’ functions, d9205 socialising.		
	Do you have difficulty watching television? (appreciating the pictures).		d110 watching		
	Do you have difficulty reading subtitles for film or TV?		d166 reading		**Seeing Functions: Participation**
	Do you have difficulty reading traffic signs, street signs, or store signs?		d166 reading		
	Do you tend to confuse colours?		b21021 colour vision		
	Do you have difficulty finding something on a crowded shelf?		b210 implicit		
**Factor 2**	How much does your eyesight interfere with using public transport on your own? (for instance bus, train or plane).		b210 ‘seeing’ functions, d4702 using public motorised transportation, e540 transportation services, systems & policies.		
	Because of my eyesight I need help from family or friends.		b210 ‘seeing’ functions, e310, e315, e320 (physical and emotional support from immediate family, extended family, friends).		**Moving Around & Communication**
	Because of my eyesight I have to rely on what other people tell me.		b210 ‘seeing’ functions, d310-329 communication - receiving (non-verbal messages, written messages).		
	Because of your eyesight, do you have difficulty going out of your home alone?		b210 ‘seeing’ functions, d4602 moving around outside the home & other buildings.		
	Do you use assistance to get around? (e.g. a guide dog, cane, companion).		d465 moving around using equipment, e115 products & technology for personal use in daily living.		
**Factor 3**	Do you worry about your eyesight getting worse?		b152 emotional function, b210 ‘seeing’ functions		
	Does your eyesight make you concerned or worried about coping with everyday life?		b152 emotional function, b210 ‘seeing’ functions		
	Do you feel like a nuisance or a burden because of your eyesight?		b152 emotional function, b210 ‘seeing’ functions		**Emotional function**
	Do you feel embarrassed because of your eyesight?		b152 emotional function, b210 ‘seeing’ functions		
	Do you feel frustrated or annoyed because of your eyesight?		b152 emotional function, b210 ‘seeing’ functions		
**Factor 4**	Because of your eyesight do you bump against other people in crowded areas?		b210 seeing functions, d4503 walking around obstacles		**Walking around obstacles**
	Do you bump into people or objects while walking?		d4503 walking around obstacles		
	Do you trip over objects?		d4503 walking around obstacles		
**Factor 5**	Does your eyesight deteriorate in bright light?		b21010 light sensitivity, e240 light (e.g. light intensity, quality and colour contrasts).		
	Does your eyesight deteriorate in dim light?		b21010 light sensitivity, e240 light.		
	Do you have difficulty with walking down steps in dim light?		d4551 climbing (e.g. climbing steps, stairs or kerbs), e240 light.		**Light**
	Do you have difficulty adjusting from bright to dim light? (such as when going from daylight into a dark room)		e240 light		
	Do you have difficulty with adjusting to bright lights?		e240 light		
**Factor 6**	Because of my eyesight I need help from care services.		b210 ‘seeing’ functions, e575 general social support services, systems & policies.		
	Because of your eyesight, do you have difficulty entertaining friends and family in your home?		b210 ‘seeing’ functions, d9205 socialising.		**Seeing Functions: Domestic & Social Life**
	When pouring liquid, do you have difficulty judging the level of the liquid in a container, such as the level of a cup of coffee?		d630 household tasks.		
	Do you have difficulty seeing how people react to things you say?		b210 ‘seeing’ functions, d3150 communication - receiving non verbal messages (body gestures)		
**Factor 7**	In the last 12 months have you been anxious or worried about falling? (This may or may not be associated with a feeling of unsteadiness).		b152 emotional function, d4 mobility (general).		**Mobility**
	Have you fallen in the last 12 months?		d410-429 changing & maintaining body position.		
	Do you have difficulty with walking on uneven ground?		d4502 walking on different surfaces.		

### Discrimination of the AGQ across glaucoma severity

**Figure 1:**
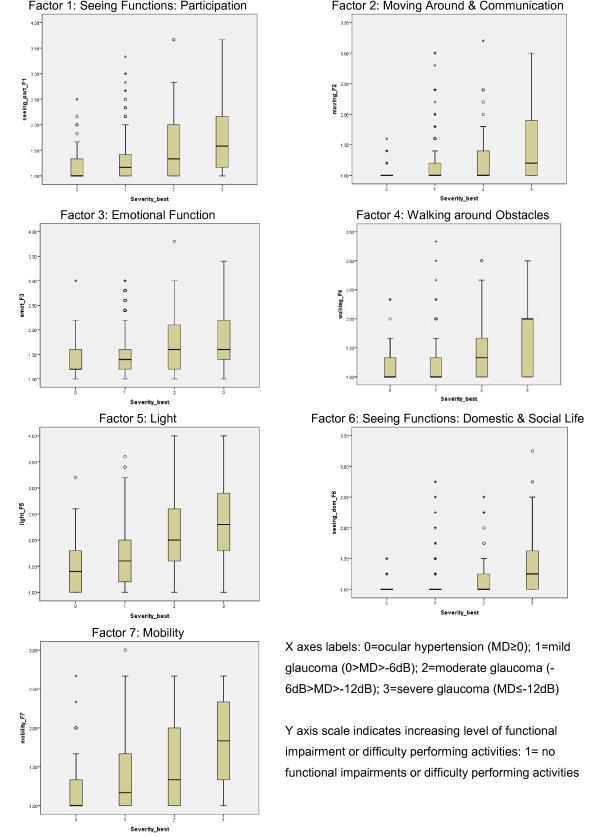
Boxplots illustrating the distribution of subscale scores by glaucoma severity (in better eye).

Seven composite subscale scores were computed (mean scores) for each participant, corresponding to the seven factors in the AGQ factor solution. Figure 1 displays the distribution of these scores by glaucoma severity in better eye and shows that as the severity of glaucoma worsens, there is increasing difficulty in performing tasks for each factor. A similar trend is displayed for the distribution of scores by glaucoma severity in the worse eye (Additional file 2). The Spearman’s rho correlations between each AGQ factor and glaucoma severity (better eye) were between 0.22 and 0.34 and all were statistically significant (p < 0.001).

The seven factor scores were entered as independent variables in a discriminant function analysis on severity of glaucoma data (ocular hypertension, mild, moderate and severe). Wilks’ Lambda indicated that the first discriminant function provided most of the separation of the groups, Chi-squared (21) = 133.89, p < 0.001. Wilks’ Lambda was non-significant for the second and third discriminant functions (all p values >0.4). The structure matrix (specifically, the correlations between each variable and the first discriminant function) was interpreted. This showed that the two most highly discriminating factors were Factor 5 (Light) and Factor 4 (Walking Around Obstacles). The profile of scores for the test of functions 1 through 3 resulted in correct classification of 32.5% of original grouped cases.

## Discussion

This paper addresses a methodological gap in the application of CTT techniques, such as PCA, in instrument development and reports a novel and reproducible method for mapping between theoretical and empirical factor structures to label empirically-derived factors. We illustrate the method by presenting results from early testing of the pilot AGQ in a population representing the spectrum of glaucoma severity using ‘real world’ visual field data collected as part of routine care.

The exploratory PCA presented a clean 7-factor (31-item) structure in which convergent and discriminant validity were evident by the high loadings within factors, relatively high Cronbach’s alphas and simple structure (no cross-loadings between factors). In addition, a conservative cut-off point of 0.5 for item inclusion was used to alleviate concern that PCA tends to underestimate the correlation when ordinal data are used. The clean structure of the 7-factor solution suggests that the 31 items in the revised AGQ cover specific aspects of glaucoma-related disability as opposed to general content about overall health or eyesight. Importantly, the empirical factor structure did not reflect the response option structure (i.e. factors contained items with different response options). We are therefore confident that the resultant factor structure arises from theoretical categories and not merely from ‘method variance’ [18].

Twenty-one items in the pilot 68 item AGQ were considered unreliable (due to >5% missing data) and were excluded from the PCA. All 3 items about difficulty with driving in different circumstances (items A55, A56, A57) were preceded by two filter questions (i.e. A52: *Have you ever driven a car?* A53: *Are you currently driving at least once in a while?*). As a result, missing data for items A55, A56 and A57 was >5% and these items were excluded from the PCA. However, people with glaucoma are known to experience difficulties driving in certain circumstances (e.g. at night) [19]. We therefore suggest that because of the difficulty including ‘activity dependent tasks’, such questions should be included alongside, but not part of the refined 7-factor, 31-item AGQ.

This paper reports how the theoretical structure produced by the systematic ICF coding of item content [3] was used to inform the labelling of the empirical factors. This innovative method not only highlighted the high level of coherence between the empirical and theoretical structure (Table 6), it also resulted in a shift in emphasis towards factor labels that reflect the potential impact of glaucoma on Activities and Participation (which are key constructs within the ICF model). For example, if looking solely at the empirical data, an appropriate label for Factor 1 (Table 4) is *Near and Distance Vision.* However, the theoretical structure of this factor (Table 6) teased out the potential impact on social participation associated with impaired vision (e.g. reading, watching television and socialising) and led to a factor label of *Seeing Functions: Participation*. Thus, this explicit method of factor labelling resulted in factor labels that have face validity, but it also addressed the lack of precision associated with subjective and atheoretical processes of labelling empirical factors. In addition, applying this method highlights the complexity of the multi-level theoretical structure of the items within a single empirical structure arising from the PCA. This variation in specificity has face validity in that it reflects the condition being investigated. In the example of the AGQ, we learn that mobility presents both general and specific challenges for people with glaucoma (i.e. the generality of the Factor 7 label; *Mobility* versus the very specific label of Factor 4; *Walking Around Obstacles*). For patient-reported outcomes relating to different conditions we would expect greater elaboration in different ICF components.

In our use of the ICF as a theoretical framework, we are taking a nuanced view of Quality of Life (QOL) and would expect the refined 7-factor (31-item) AGQ to be able to distinguish associations between factor (subscale) scores and different kinds of QOL vulnerability. However, the most appropriate scoring method and the validity of using the seven empirical factors as subscales are questions for further research.

A secondary objective of this paper was to report findings on the discriminative ability of the AGQ. In Figure 1 the boxplots indicate a clear signal in the data. For example, visual inspection of the boxplots for the Factors *Walking Around Obstacles; Light; Mobility* strongly suggest that as the severity of glaucoma gets worse, there is increasing difficulty in performing tasks. However, it must be noted that for all 7 factors, the signal is masked by high within-group variability. The observed trend was similar for the boxplots illustrating the distribution of subscale scores by glaucoma severity in the worse eye (Additional file 2), although the signal appears weaker. The results of the discriminant function analysis suggest that the AGQ performs significantly better than chance in classifying respondents’ glaucoma severity, and performs best discriminating between those with moderate to severe glaucoma based on the visual field loss in the better eye. However, the use of scores for identifying individual-level severity would not be appropriate.

The study used routine data collected in clinical care. It was not set up to examine all patients in terms of their clinical glaucoma severity and therefore misclassification of cases as ocular hypertension or glaucoma could have occurred. However, the use of the Glaucoma hemifield test to filter for likely glaucoma would minimise misclassification. Future research could test the discriminant ability of the AGQ in a prospective study including a clinical classification of ocular hypertension and glaucoma.

In our current analysis we evaluated visual field loss based on MD data for each participant’s better eye (with an additional exploratory analysis for worse eye), rather than using a binocular visual field measure. One potential limitation of this approach is that for individual patients, areas of vision loss in each eye may not necessarily overlap and visual field data for separate eyes may not capture the person’s binocular visual field. In addition, the analysis does not include validation of the measures, AGQ or NEIVFQ, against location of the visual field defect as within the design of this study we only had access to the MD a global index of visual field loss. Location of visual field loss is likely to relate to the severity of any patient reported disability. This is an important area for future research.

Another limitation of the study is the potential for response bias, given a 49% response rate. We were restricted by the study ethics approval from including copies of the questionnaires with the single reminder letter to non-responders and from obtaining visual field data on non-responders. Thus the effectiveness of the reminder letter on increasing the response rate was reliant on patients having retained the original copy of the AGQ. The lack of visual field data on non-responders meant we were unable to compare the demographic characteristics and level of glaucoma severity of responders and non-responders. Despite these limitations, the 49% response rate is higher than generally achieved in postal surveys of glaucoma patients [20] and to our knowledge this is the largest study of its kind.

This paper presents a robust method for linking the empirical factors with a theoretical factor structure and for assigning theoretically informed labels during early testing of new PRO instruments using CTT. We recommend that further testing (e.g. Rasch analysis) be conducted to provide greater insight into the psychometric properties and dimensionality of the AGQ. Such testing will aid interpretation of the AGQ as a measure of vision-related disability in glaucoma patients and inform item refinement [21].

## Conclusions

This is the first step in a series of studies that will progressively assess the validity and utility of the AGQ. Several glaucoma-specific PRO measures are available, however none have linked the empirical factor structure to the ICF model and are thus not transparent about the constructs of health status measured [2]. In addition, factor labels are often selected intuitively whereas they can inform existing bodies of knowledge if selected on the basis of theoretical construct labels, which are more explicitly defined and which relate to each other in ways that are evidence based. Our approach illustrates a new method in which decisions of what constitutes an empirically-derived factor were driven jointly by statistical evidence (using a higher than usual cut-off point of 0.5) and the theoretical ICF structure established during the pre-validation development of the AGQ.

A secondary objective of this study was to explore the ability of the AGQ to discriminate between people without glaucoma and those with significant disease. The data support the validity of the AGQ in this context. Our findings are based on responses from a hospital-based sample. The AGQ is now ready for further testing in a more general population setting including those at risk of, but not necessarily with, established glaucoma.

## Abbreviations

PRO: Patient reported outcome; PCA: Principal components analysis; AGQ: Aberdeen Glaucoma Questionnaire; ICF: International classification of functioning disability and health; MEH: Moorfields Eye Hospital; MD: Mean defect; QOL: Quality of life.

## Competing interests

The authors declare they have no competing interests.

## Authors’ contributions

JB, CR, JF were grant holders and designed the study. All authors were actively involved in the study working group. CR and DJ conducted the analysis. MP, JF, JB and CR wrote early drafts of the manuscript. All authors commented on, revised and approved the final version of the manuscript.

## Pre-publication history

The pre-publication history for this paper can be accessed here:

http://www.biomedcentral.com/1471-2415/13/72/prepub

## Supplementary Material

Additional file 1 The AGQ. Click here for file

Additional file 2 Boxplots illustrating the distribution of subscale scores by glaucoma severity (in worse eye). Click here for file
