# Supplementary material for: Theoretical and empirical dimensions of the Aberdeen Glaucoma Questionnaire: a cross sectional survey and principal component analysis
Source: BMC Ophthalmol. 2013 Nov 22;13:72. doi: 10.1186/1471-2415-13-72 (PMC4222643; doi:10.1186/1471-2415-13-72)
Supplement: Additional file 1 — The AGQ. [file 1471-2415-13-72-S1.pdf]

Study No

VISION QUESTIONNAIRE

The questions ask you about your eyesight, general health and everyday life activities. We would be grateful if you could complete and return this questionnaire.

**If you require the questionnaire in larger text or if you would like any further information or have any questions, please contact:
Susan Campbell, tel: 01224 559023**

Thank you for taking the time to help us with this study.

CONFIDENTIAL

Version 2, February 2010

HOW TO FILL IN THIS QUESTIONNAIRE:

The questions can be answered by putting a tick (✓) in the appropriate box or boxes. If you make any errors while completing the form, shade out the box completely and place a tick in the appropriate box.

If you wear glasses or contact lenses, please answer all of the following questions as though you were wearing them.

STATEMENT OF CONFIDENTIALITY:

All information that would permit identification of any person who completed this questionnaire will be regarded as strictly confidential. Such information will be used only for the purposes of this study and will not be disclosed or released for any other purposes without prior consent, except as required by law.

SECTION A – ABOUT YOUR EYESIGHT TODAY

A1. Does your eyesight deteriorate in bright light?

Not at all ☐

A little ☐

Quite a lot ☐

Very much ☐

A2. Does your eyesight deteriorate in dim light?

Not at all ☐

A little ☐

Quite a lot ☐

Very much ☐

A3. Does your eyesight interfere with your recognising or meeting people?

No ☐

Sometimes ☐

Often ☐

Always ☐

A4. Because of your eyesight do you bump against other people in crowded areas?

No ☐

Sometimes ☐

Often ☐

Always ☐

A5. Have you had to give up any activities because of your eyesight?

- No ☐
- Uncertain ☐
- Yes ☐

A6. Do you worry about your eyesight getting worse?

- Not at all ☐
- A little of the time ☐
- A lot of the time ☐
- All of the time ☐

A7. Does your eyesight make you concerned or worried about coping with everyday life?

- Not at all ☐
- A little of the time ☐
- A lot of the time ☐
- All of the time ☐

A8. How often does your eyesight stop you doing the things you want to do?

- Not at all ☐
- A little of the time ☐
- A lot of the time ☐
- All of the time ☐

A9. **Do you feel like a nuisance or a burden because of your eyesight?**

Not at all ☐

A little of the time ☐

A lot of the time ☐

All of the time ☐

A10. **Do you feel embarrassed because of your eyesight?**

Not at all ☐

A little of the time ☐

A lot of the time ☐

All of the time ☐

A11. **Do you feel frustrated or annoyed because of your eyesight?**

Not at all ☐

A little of the time ☐

A lot of the time ☐

All of the time ☐

A12. **How much does your eyesight interfere with your getting about outdoors? (on the pavement or crossing the street)**

Not at all ☐

A little of the time ☐

A lot of the time ☐

All of the time ☐

A13. How much does your eyesight interfere with generally looking after your appearance? (face, hair, clothing etc.)

- Not at all ☐
- A little of the time ☐
- A lot of the time ☐
- All of the time ☐

A14. How much does your eyesight make you concerned or worried about spilling or breaking things?

- Not at all ☐
- A little of the time ☐
- A lot of the time ☐
- All of the time ☐

A15. How much does your eyesight interfere with using public transport on your own? (for instance bus, train or plane)

- Not at all ☐
- A little of the time ☐
- A lot of the time ☐
- All of the time ☐

A16. Because of my eyesight I need help from family or friends.

- Not at all ☐
- A little of the time ☐
- A lot of the time ☐
- All of the time ☐

A17. Because of my eyesight I need help from care services.

- Not at all ☐
- A little of the time ☐
- A lot of the time ☐
- All of the time ☐

A18. Because of my eyesight I have to rely on what other people tell me.

- Not at all ☐
- A little of the time ☐
- A lot of the time ☐
- All of the time ☐

A19. Because of your eyesight, do you have difficulty going out of your home alone?

- No ☐
- A little ☐
- Moderate ☐
- Extreme ☐
- I am unable to go out alone ☐

A20. Because of your eyesight, do you have difficulty entertaining friends and family in your home?

- No ☐
- A little ☐
- Moderate ☐
- A great deal ☐
- I am unable to do the activity ☐
- Not applicable (I never do this activity) ☐

A21. Does you eyesight interfere with your going to sports events, plays or films?

- No ☐
- A little ☐
- Moderately ☐
- A great deal ☐
- I am unable to do the activity ☐
- Not applicable (I never go to such events) ☐

A22. Do you have difficulty walking in dimly lit indoor areas?

- No ☐
- A little ☐
- Moderate ☐
- Extreme ☐
- I am unable to walk ☐

A23. **Do you have difficulty with walking on uneven ground?**

No ☐

A little ☐

Moderate ☐

Extreme ☐

I am unable to walk ☐

A24. **Do you have difficulty with walking down steps in dim light?**

No ☐

A little ☐

Moderate ☐

Extreme ☐

I am unable to walk ☐

A25. **Do you have difficulty doing any type of work which requires you to see well up close?**

No ☐

A little ☐

Moderate ☐

Extreme ☐

A26. **In the last 12 months have you been anxious or worried about falling? (This may or may not be associated with a feeling of unsteadiness)**

No ☐

Yes ☐

A27. Have you fallen in the last 12 months?

No ☐

Yes ☐

If 'Yes', how many times ☐

A28. Do you bump into people or objects while walking?

No ☐

Sometimes ☐

Often ☐

Always ☐

A29. Do you trip over objects?

No ☐

Sometimes ☐

Often ☐

Always ☐

A30. **Do you use assistance to get around? (e.g. a guide dog, cane, companion)**

No ☐

Sometimes ☐

Often ☐

Always ☐

A31. **When you reach for an object, do you find that it is further away, or closer, than you think?**

Never ☐

Sometimes ☐

Often ☐

Always ☐

A32. **Do objects ever suddenly appear when you should have noticed them before?**

No ☐

Uncertain ☐

Yes ☐

A33. **Do you have difficulty making out differences in coins and notes?**

No ☐

A little ☐

Moderate ☐

Extreme ☐

A34. **Do you have difficulty filling out forms or writing cheques?**

No ☐

A little ☐

Moderate ☐

Extreme ☐

A35. **When pouring liquid, do you have difficulty judging the level of the liquid in a container, such as the level of a cup of coffee?**

No ☐

A little ☐

Moderate ☐

Extreme ☐

A36. **Do you have difficulty finding something on a crowded shelf?**

No ☐

A little ☐

Moderate ☐

Extreme ☐

A37. **Do you have difficulty seeing well enough to do manual activities such as cooking, sewing, cutting your nails?**

No ☐

A little ☐

Moderate ☐

Extreme ☐

A38. Do you have difficulty seeing how people react to things you say?

- No ☐
- A little ☐
- Moderate ☐
- Extreme ☐

A39. Do you have difficulty adjusting from bright to dim light? (such as when going from daylight into a dark room)

- No ☐
- A little ☐
- Moderate ☐
- Extreme ☐

A40. Do you have difficulty with adjusting to bright lights?

- No ☐
- A little ☐
- Moderate ☐
- Extreme ☐

A41. Do you have difficulty watching television? (appreciating the pictures)

- No ☐
- A little ☐
- Moderate ☐
- Extreme ☐

A42. Do you have difficulty reading subtitles for film or TV?

- No ☐
- A little ☐
- Moderate ☐
- Extreme ☐

A43. Do you have difficulty reading small print under poor lighting?

- No ☐
- A little ☐
- Moderate ☐
- Extreme ☐

A44. Do you have difficulty reading traffic signs, street signs, or store signs?

- No ☐
- A little ☐
- Moderate ☐
- Extreme ☐

A45. Do you tend to confuse colours?

- No ☐
- Sometimes ☐
- Often ☐
- Always ☐

A46. Do you have difficulty playing games such as bingo, dominos or card games?

- No ☐
- A little ☐
- Moderate ☐
- A great deal ☐
- I am unable to do the activity ☐
- Not applicable (I never play these types of games) ☐

A47. Do you have difficulty seeing steps or kerbs?

- No ☐
- A little ☐
- Moderate ☐
- Extreme ☐

A48. Do you notice that parts of your vision are missing?

- No ☐
- Yes ☐

A49. Do you feel lonely?

- Not at all ☐
- A little of the time ☐
- A lot of the time ☐
- All of the time ☐

A50. Are you experiencing any of the following?

	No	Yes
Eyes watering	☐	☐
Redness in and around your eyes	☐	☐
Lengthening of eye lashes	☐	☐
Pain or discomfort in and around your eyes	☐	☐
Burning or stinging in and around your eyes	☐	☐
Itching in and around your eyes	☐	☐
Colour changes in your eyes and to the skin around your eyes	☐	☐

A51. Are you experiencing any of the following?

	No	Yes
Headache	☐	☐
Shortness of breath	☐	☐
Tiredness	☐	☐
Bitter taste in the mouth	☐	☐
Unusual taste in the mouth	☐	☐
Sexual problems	☐	☐

A52. Have you ever driven a car?

No	☐	Go to SECTION B PAGE 18
Yes	☐	Go to A53

A53. Are you currently driving at least once in a while?

No	☐	Go to A54
Yes	☐	Go to A55

A54. Why did you stop driving?

Vision	☐	Go to SECTION B PAGE 18
Other illness	☐	Go to SECTION B
Other reason	☐	Go to SECTION B

A55. Do you have difficulty driving in unfamiliar places?

- No difficulty at all ☐
- A little difficulty ☐
- Moderate difficulty ☐
- Extreme difficulty ☐

A56. Do you have difficulty driving in familiar places?

- No difficulty at all ☐
- A little difficulty ☐
- Moderate difficulty ☐
- Extreme difficulty ☐

A57. Do you have difficulty driving at night?

- No ☐
- A little ☐
- Moderate ☐
- Extreme ☐
- I am unable to drive at night ☐

SECTION B– CAN YOU PLEASE TELL US A LITTLE BIT ABOUT YOURSELF?

B1. **Are you male or female?**

Male ☐

Female ☐

B2. **What is your age?** ______________________

B3. **In general, would you say your health is?**

Excellent ☐

Very good ☐

Good ☐

Fair ☐

Poor ☐

SECTION C – GENERAL HEALTH TODAY

The next section is about your health in **general**. By placing a tick in one box in each group below, please indicate which statements best describe your own health state **today**.

C1. **Mobility**

I have no problem in walking about ☐

I have some problems in walking about ☐

I am confined to bed ☐

C2. **Self-care**

I have no problems with self-care ☐

I have some problems washing myself or dressing myself ☐

I am unable to wash or dress myself ☐

C3. **Usual activities (such as work, study, housework, family or leisure activities)**

I have no problems with performing my usual activities ☐

I have some problems with performing my usual activities ☐

I am unable to perform my usual activities ☐

C4. **Pain/discomfort**

I have no pain or discomfort ☐

I have moderate pain or discomfort ☐

I have extreme pain or discomfort ☐

C5. **Anxiety/depression**

I am not anxious or depressed ☐

I am moderately anxious or depressed ☐

I am extremely anxious or depressed ☐

DESCRIBING YOUR OWN HEALTH TODAY

Please indicate on this scale how good or bad your own health state is today.

The best health state you can imagine is marked 100 and the worst health state you can imagine is marked 0.

Please draw a line from box A to the point on the scale that best indicates how good or bad your health state is today.

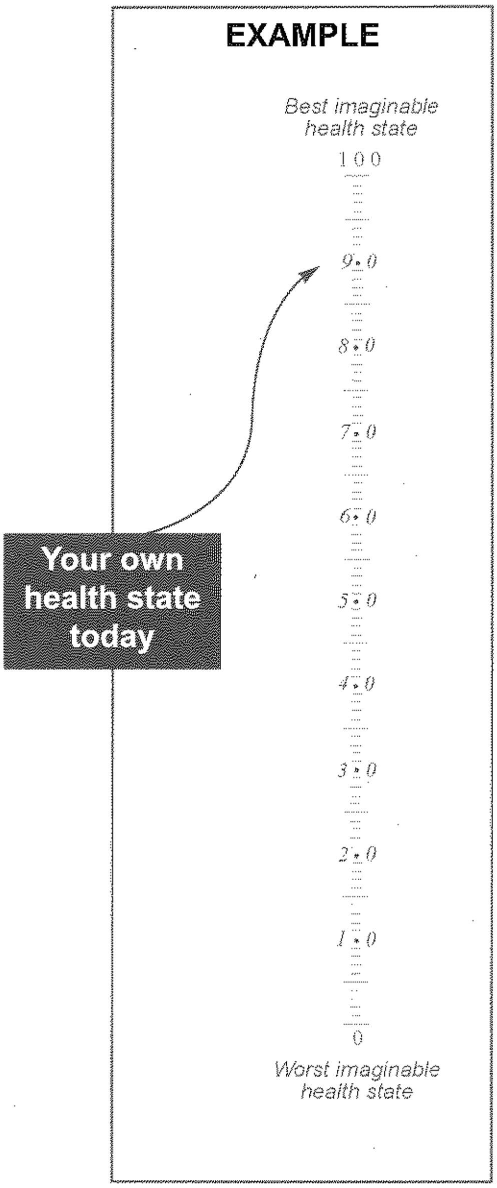

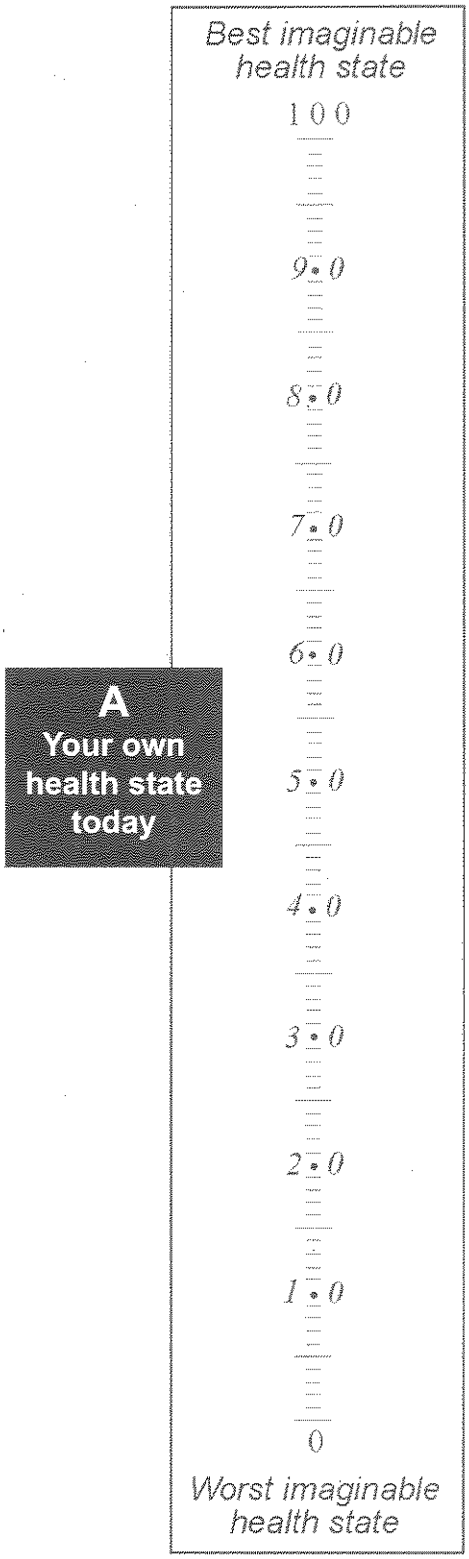

SECTION D – VISUAL FUNCTIONING QUESTIONNAIRE – (NEI-VFQ-25)

The following is a survey with statements about problems, which involve your vision, or feelings that you have about your eye condition. After each question please choose the response that best describes your situation. Please take as much time as you need to answer each question. All your answers are confidential. In order for this survey to improve our knowledge about eye problems and how they affect your quality of life, your answers must be as accurate as possible. Remember, if you wear glasses or contact lenses, please answer all of the following questions as though you were wearing them.

INSTRUCTIONS:

1. In general we would like to have people try to complete these forms on their own. If you find that you need assistance, please feel free to ask a friend or relative to assist you, or phone the project office on 01224 559023

2. Please answer every question (unless you are asked to skip questions because they don't apply to you).

3. Answer the questions by circling the appropriate number.

4. If you are unsure of how to answer a question, please give the best answer you can and make a comment in the left margin.

© R 1996

PART 1 – GENERAL HEALTH AND VISION

1. **In general, would you say your overall health is:**
(Circle One)

Excellent	1
Very Good	2
Good	3
Fair	4
Poor	5

2. **At the present time, would you say your eyesight using both eyes (with glasses or contact lenses, if you wear them) is excellent, good, fair, poor, or very poor or are you completely blind?**
(Circle One)

Excellent	1
Good	2
Fair	3
Poor	4
Very Poor	5
Completely Blind	6

3. **How much of the time do you worry about your eyesight?**
(Circle One)

None of the time	1
A little of the time	2
Some of the time	3
Most of the time	4
All of the time	5

4. **How much pain or discomfort have you had in and around your eyes (for example, burning, itching, or aching)? Would you say it is:**
(Circle One)

None	1
Mild	2
Moderate	3
Severe, or	4
Very severe	5

PART 2 – DIFFICULTY WITH ACTIVITIES

The next questions are about how much difficulty, if any, you have doing certain activities wearing your glasses or contact lenses if you use them for that activity.

5. **How much difficulty do you have reading ordinary print in newspapers? Would you say you have:**
(Circle One)

No difficulty at all	1
A little difficulty	2
Moderate difficulty	3
Extreme difficulty	4
Stopped doing this because of your eyesight	5
Stopped doing this for other reasons or not interested in doing this	6

6. **How much difficulty do you have doing work or hobbies that require you to see well up close, such as cooking, sewing, fixing things around the house, or using hand tools? Would you say:**
(Circle One)

No difficulty at all	1
A little difficulty	2
Moderate difficulty	3
Extreme difficulty	4
Stopped doing this because of your eyesight	5
Stopped doing this for other reasons or not interested in doing this	6

7. **Because of your eyesight, how much difficulty do you have finding something on a crowded shelf?**
(Circle One)

No difficulty at all	1
A little difficulty	2
Moderate difficulty	3
Extreme difficulty	4
Stopped doing this because of your eyesight	5
Stopped doing this for other reasons or not interested in doing this	6

8. How much difficulty do you have reading street signs or the names of stores?
(Circle One)

No difficulty at all	1
A little difficulty	2
Moderate difficulty	3
Extreme difficulty	4
Stopped doing this because of your eyesight	5
Stopped doing this for other reasons or not interested in doing this	6

9. Because of your eyesight, how much difficulty do you have going down steps, stairs, or curbs in dim light or at night?
(Circle One)

No difficulty at all	1
A little difficulty	2
Moderate difficulty	3
Extreme difficulty	4
Stopped doing this because of your eyesight	5
Stopped doing this for other reasons or not interested in doing this	6

10. Because of your eyesight, how much difficulty do you have noticing objects off to the side while you are walking along?
(Circle One)

No difficulty at all	1
A little difficulty	2
Moderate difficulty	3
Extreme difficulty	4
Stopped doing this because of your eyesight	5
Stopped doing this for other reasons or not interested in doing this	6

11. Because of your eyesight, how much difficulty do you have seeing how people react to things you say?
(Circle One)

No difficulty at all	1
A little difficulty	2
Moderate difficulty	3
Extreme difficulty	4
Stopped doing this because of your eyesight	5
Stopped doing this for other reasons or not interested in doing this	6

12. **Because of your eyesight, how much difficulty do you have picking out and matching your own clothes?**
(Circle One)

No difficulty at all	1
A little difficulty	2
Moderate difficulty	3
Extreme difficulty	4
Stopped doing this because of your eyesight	5
Stopped doing this for other reasons or not interested in doing this	6

13. **Because of your eyesight, how much difficulty do you have visiting with people in their homes, at parties, or in restaurants?**
(Circle One)

No difficulty at all	1
A little difficulty	2
Moderate difficulty	3
Extreme difficulty	4
Stopped doing this because of your eyesight	5
Stopped doing this for other reasons or not interested in doing this	6

14. **Because of your eyesight, how much difficulty do you have going out to see movies, plays, or sports events?**
(Circle One)

No difficulty at all	1
A little difficulty	2
Moderate difficulty	3
Extreme difficulty	4
Stopped doing this because of your eyesight	5
Stopped doing this for other reasons or not interested in doing this	6

15. **Are you currently driving, at least once in a while?**
(Circle One)

Yes	1	*Skip To Q 15c*
No	2	

15a. **IF NO: Have you never driven a car or have you given up driving?**
(Circle One)

Never drove	1	*Skip To Part 3, Q 17*
Gave up	2	

15b. **IF YOU GAVE UP DRIVING: Was that mainly because of your eyesight, mainly for some other reason, or because of both your eyesight and other reasons?**
(Circle One)

Mainly eyesight	1	*Skip To Part 3, Q 17*
Mainly other reasons	2	*Skip To Part 3, Q 17*
Both eyesight and other reasons	3	*Skip To Part 3, Q 17*

15c. **IF CURRENTLY DRIVING: How much difficulty do you have driving during the daytime in familiar places? Would you say you have:**
(Circle One)

No difficulty at all	1
A little difficulty	2
Moderate difficulty	3
Extreme difficulty	4

16. **How much difficulty do you have driving at night? Would you say you have:**
(Circle One)

No difficulty at all	1
A little difficulty	2
Moderate difficulty	3
Extreme difficulty	4
Stopped doing this because of your eyesight	5
Stopped doing this for other reasons or not interested in doing this	6

16a. **How much difficulty do you have driving in difficult conditions, such as in bad weather, during rush hour, on the freeway, or in city traffic? Would you say you have:**
(Circle One)

No difficulty at all	1
A little difficulty	2
Moderate difficulty	3
Extreme difficulty	4
Stopped doing this because of your eyesight	5
Stopped doing this for other reasons or not interested in doing this	6

PART 3 – RESPONSES TO VISION PROBLEMS

The next questions are about how things you do may be affected by your vision. For each one, please circle the number to indicate whether for you the statement is true for you all, most, some, a little, or none of the time.

(Circle One On Each Line)

**READ CATEGORIES:**	All of the time	Most of the time	Some of the time	A little of the time	None of the time
17. Do you accomplish less than you would like because of your vision?	1	2	3	4	5
18. Are you limited in how long you can work or do other activities because of your vision?	1	2	3	4	5
19. How much does pain or discomfort in or around your eyes, for example, burning, itching, or aching, keep you from doing what you'd like to be doing? Would you say:	1	2	3	4	5

For each of the following statements, please circle the number to indicate whether for you the statement is definitely true, mostly true, mostly false, or definitely false for you or you are not sure.
(Circle One On Each Line)

**READ CATEGORIES:**	Definitely True	Mostly True	Not Sure	Mostly False	Definitely False
20. I stay home most of the time because of my eyesight	1	2	3	4	5
21. I feel frustrated a lot of the time because of my eyesight.	1	2	3	4	5
22. I have much less control over what I do, because of my eyesight.	1	2	3	4	5
23. Because of my eyesight, I have to rely too much on what other people tell me...	1	2	3	4	5
24. I need a lot of help from others because of my eyesight.	1	2	3	4	5
25. I worry about doing things that will embarrass myself or others, because of my eyesight.	1	2	3	4	5

Date you filled in this questionnaire ☐☐ / ☐☐ / ☐☐☐☐

Have you needed help to fill this questionnaire? Yes ☐ No ☐

THANK YOU

Thank you very much for your time and patience in filling in this questionnaire.

The information you have given us will be extremely useful in helping us carry out our research into treatments for Glaucoma. It will be treated with the strictest confidence and kept securely.

Thank you again for your help

If you would like any further information or have any queries about the study, please contact:

GPS Glaucoma Screening Platform Study

GPS Study Office
Health Services Research Unit
University of Aberdeen
3rd Floor, Health Sciences Building
Foresterhill Road
Aberdeen
AB25 2ZD
Tel: 01224 559023
