# Supplementary material for: Theoretical and empirical dimensions of the Aberdeen Glaucoma Questionnaire: a cross sectional survey and principal component analysis
Source: BMC Ophthalmol. 2013 Nov 22;13:72. doi: 10.1186/1471-2415-13-72 (PMC4222643; doi:10.1186/1471-2415-13-72)
Supplement: Additional file 2 — Boxplots illustrating the distribution of subscale scores by glaucoma severity (in worse eye). [file 1471-2415-13-72-S2.pdf]

Additional File 2:

Boxplots illustrating the distribution of subscale scores by glaucoma severity (in worse eye)

Factor 1: Seeing Functions: Participation

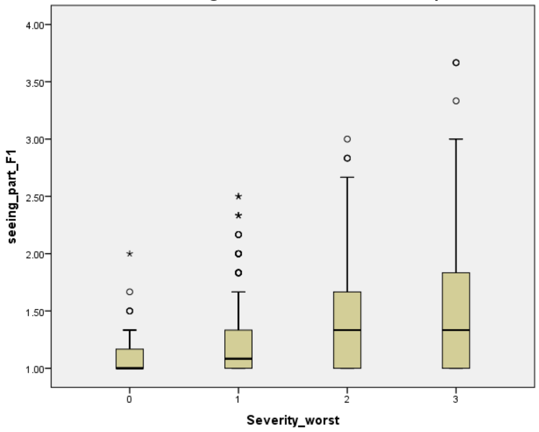

Factor 2: Moving Around & Communication

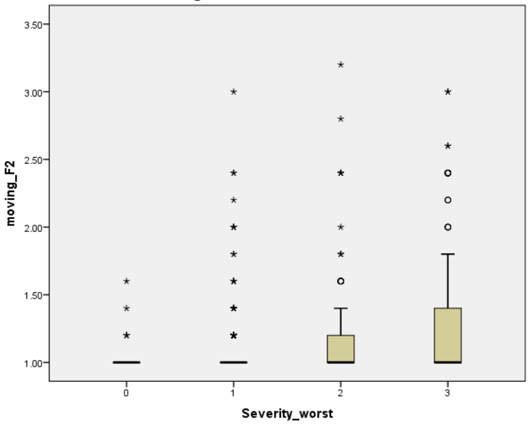

Factor 3: Emotional Function

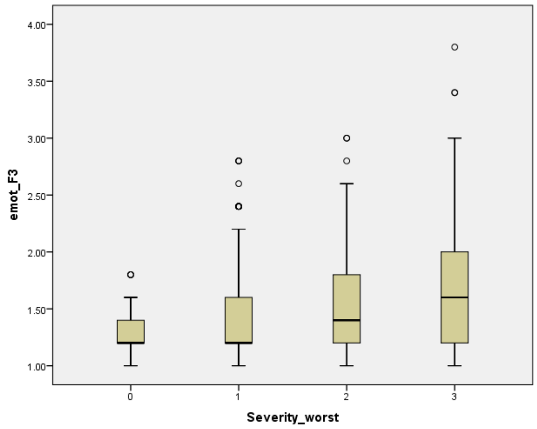

Factor 4: Walking around Obstacles

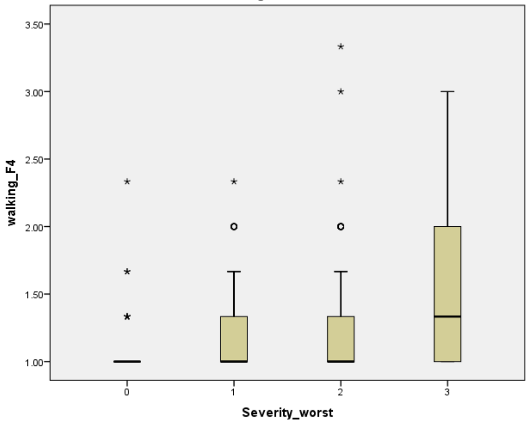

Factor 5: Light

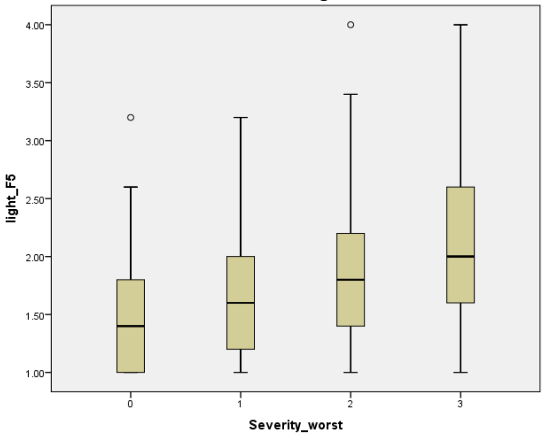

Factor 6: Seeing Functions: Domestic & Social Life

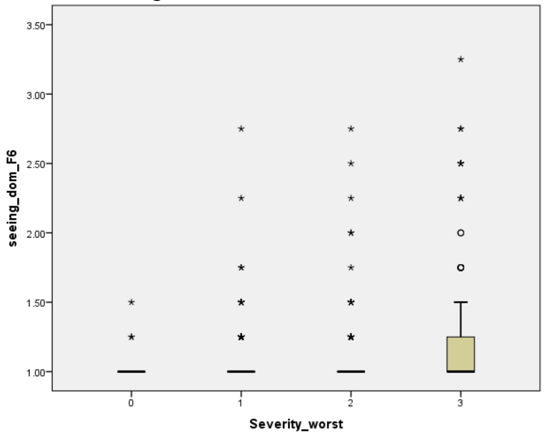

Factor 7: Mobility

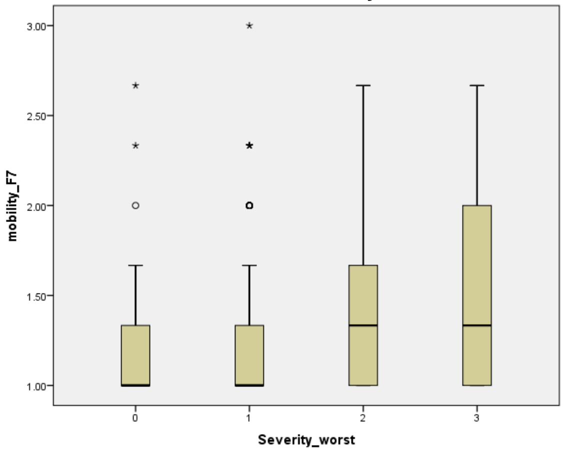

X axes labels: 0=ocular hypertension (MD≥0); 1=mild glaucoma (0>MD>-6dB); 2=moderate glaucoma (-6dB>MD>-12dB); 3=severe glaucoma (MD≤-12dB)

Y axis scale indicates increasing level of functional impairment or difficulty performing activities: 1= no functional impairments or difficulty performing activities
